# The Effect of Glycerol, Sugar, and Maleic Anhydride on Pectin-Cellulose Thin Films Prepared from Orange Waste

**DOI:** 10.3390/polym11030392

**Published:** 2019-02-27

**Authors:** Veronika Bátori, Magnus Lundin, Dan Åkesson, Patrik R. Lennartsson, Mohammad J. Taherzadeh, Akram Zamani

**Affiliations:** Swedish Centre for Resource Recovery, University of Borås, 50190 Borås, Sweden; magnus.lundin@hb.se (M.L.); dan.akesson@hb.se (D.Å.); patrik.lennartsson@hb.se (P.R.L.); mohammad.taherzadeh@hb.se (M.J.T.); akram.zamani@hb.se (A.Z.)

**Keywords:** bio-based, film, mechanical properties, polysaccharides, resource recovery, solution casting, orange waste

## Abstract

This study was conducted to improve the properties of thin films prepared from orange waste by the solution casting method. The main focus was the elimination of holes in the film structure by establishing better cohesion between the major cellulosic and pectin fractions. For this, a previously developed method was improved first by the addition of sugar to promote pectin gelling, then by the addition of maleic anhydride. Principally, maleic anhydride was introduced to the films to induce cross-linking within the film structure. The effects of concentrations of sugar and glycerol as plasticizers and maleic anhydride as a cross-linking agent on the film characteristics were studied. Maleic anhydride improved the structure, resulting in a uniform film, and morphology studies showed better adhesion between components. However, it did not act as a cross-linking agent, but rather as a compatibilizer. The middle level (0.78%) of maleic anhydride content resulted in the highest tensile strength (26.65 ± 3.20 MPa) at low (7%) glycerol and high (14%) sugar levels and the highest elongation (28.48% ± 4.34%) at high sugar and glycerol levels. To achieve a uniform film surface with no holes present, only the lowest (0.39%) level of maleic anhydride was necessary.

## 1. Introduction

The post-plastic era induces several industries, such as the biomedical, building, and packaging industries, to move towards the use of bio-based films. Thin films prepared from lignocellulosic by-products of food and agricultural industries not only have a great potential to replace petroleum-based films, but the recovery of biopolymers also forms an essential part of the bioeconomy [1]. Cellulose, in particular, is abundant and is a favorable reinforcement material because of its features, such as its crystalline structure and good mechanical properties [2]. 

An attractive research area is the production of bio-based films from fruit and vegetable residues, often with the film-casting method [3,4,5,6,7,8]. During film-casting, the suspension of a colloidal dispersion of polymers is poured onto a non-sticky surface and dried to a film. Various fruit and vegetable residuals are rich sources of different biopolymers, such as pectin, starch, cellulose, hemicellulose, lignin, and proteins, which can be used to form firm films with properties rivaling those of commodity plastics. By choosing the right additives, biopolymers can be dissolved, plasticized, or kept undissolved in order to perform the required function in the bio-based film, i.e. turned into a matrix, blended, or used as reinforcement. One study [6], for example, reported that in bio-based films made from carrot powders, the insoluble, mostly crystalline cellulose fractions were responsible for the film structure, while the soluble, mostly pectin and sugar phase had a plasticizing effect.

When fabricating bioplastics from polysaccharides, one has to bear in mind that these are hydrophobic substances that have low water vapor barrier properties [9] and that the mechanical and barrier properties are affected by moisture content. Several methods exist to enhance the barrier and mechanical properties of hydrocolloid films; one promising alternative is the modification of physical properties by inducing intra-, and intermolecular bonding by chemical, enzymatic, or physical cross-linking [10]. Formaldehyde, glutaraldehyde, and glyoxal are potent cross-linking agents that enhance water barrier properties and increase the strength of edible protein-based films [11]. Due to their toxicity, however, their use is limited for environmentally friendly applications. A low hazard profile compound and cross-linking agent is maleic anhydride [12,13]. Maleic anhydride has also been used as a compatibilizer between natural fibers and poly(lactic acid) matrices [14,15] to improve adhesion.

Bio-based films from the by-product of industrial orange juice processing, mostly containing peels, seeds, pulp, and membrane residues, have previously been developed with suitable mechanical properties, such as a tensile strength ranging between 27.3 and 36.7 MPa [8]. Briefly, a citric acid solution containing orange waste (composed mainly of pectin and cellulosic fibers) powder and glycerol was casted for bio-based film production. Due to the mechanism of drying of colloidal films, a number of holes were present in the structure, however. In the same study, the use of a rotary movement during drying showed positive effects, but film thicknesses were uneven, and the continuous rotary movement had a higher energy consumption. To deal with this unfavorable aspect, further improvement in the preparation conditions and structure of the films was necessary. 

Thus, this study was dedicated first to investigating the enhancement of pectin gelling, establishing a three-dimensional network in which other components are trapped [16]. To achieve this goal, conditions for solubilizing pectin first had to be provided, followed by conditions that allow pectin gelling. The reason for this was the fact that within conditions for gelling, solubilization would not happen, and vice versa. This meant that the addition of citric acid (acid being one of the triggers for pectin gelling), together with sugar, took place in a later stage to promote pectin gelling [16]. This step aimed to reduce the holes by establishing an improved structure of pectin. This study also investigated the use of maleic anhydride to improve the properties of orange waste pectin–cellulose thin films. Maleic anhydride has been used previously to develop maleic anhydride-grafted orange waste [17]; therefore, it was expected that it would have a cross-linking effect on the films. Mechanical tests and morphological studies were performed to investigate the effect of each component and optimize the preparation conditions.

## 2. Materials and Methods

### 2.1. Materials

Orange waste, obtained in the juice production process and consisting of mostly pectin, hemicelluloses, and cellulose [8], was kindly provided by Brämhults AB (Borås, Sweden) before the headquarters changed location, and was kept at −20 °C until further use. Maleic anhydride (≥99%, Sigma Aldrich, St. Louise, MO, USA), citric acid (monohydrate, >99.5%, Duchefa Biochemie, Haarlem, the Netherlands), d-(+)-glucose (≥99.5%, Sigma Aldrich, St. Louise, MO, USA), herein referred to as sugar, and glycerol (>99 % ARCOS Organics, Antwerp, Belgium) were other materials used in the experiments.

### 2.2. Pretreatment of Orange Waste

Pretreatment of orange waste was performed according to a previous study [17] in which soluble sugars were removed prior to size reduction, drying, and milling of the material to a fine powder. In the first step of milling, a variable speed rotor mill (Pulverisette 14, Fritsch, Idar-Oberstein, Germany) was used with a sequence of sieve sizes of 1 and 0.2 mm at 10,000 rpm for a maximum of 1 min each. The powder was further milled with a ball-mill (MM 400, Retsch, Haan, Germany) at a frequency of 30 Hz for 10 min. The second step of milling was necessary in order to obtain the same range of particles (mostly between 125 and 75 µm) as in a previous study [8].

### 2.3. Film Formation

Film formation of orange waste was further developed from the method applied in our previous study [8]. The first step of the modification was done by applying a “sol-gel method”. In the sol-gel method, first, conditions for solubilizing the pectin are provided, followed by conditions for gelling the pectin [16]. By this method, 2% (*w*/*v*) orange waste was introduced to 100 mL of vigorously stirred distilled water that already contained 7% glycerol (*w*/*w* of orange waste powder) and 1 drop of organic antifoam (Antifoam 204, Sigma Aldrich, St. Louise, MO, USA) at 40–50 °C. The mixture was then heated up to 70 °C and cooled to about 60 °C. At that point, citric acid (to obtain a 1% (*w*/*v*) solution) and 7% sugar (*w*/*w* of orange waste powder) were added to the mixture to initiate the gelling, and rotation was reduced to 200 rpm to support pectin gelling. Then, 30 g of the suspension was poured through a metal sieve, to capture occasionally formed air bubbles, onto non-sticky polytetrafluoroethylene plates and dried in a laboratory oven (Termaks, TS9026, Bergen, Norway) at 40 °C.

**Figure 1:**
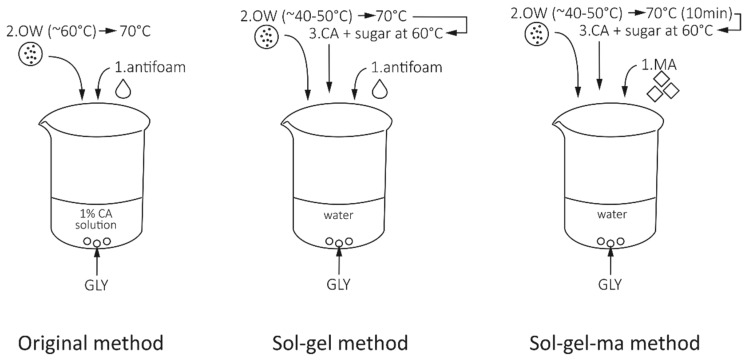
Schematic image of the different film-forming methods discussed in this study. Glycerol (GLY), orange waste (OW), citric acid (CA), and maleic anhydride (MA) were ingredients used in the methods.

The “sol-gel-ma method” was a further step in which maleic anhydride, as a potential crosslinking agent, was also incorporated into the recipe of the sol-gel method, while antifoam was removed to reduce the number of chemicals. In the sol-gel-ma method, different concentrations of maleic anhydride (100, 50, 25, 12.5, 6.25, 3.13, and 1.56 (*w*/*w*) %) were used in the preliminary experiments, and maleic anhydride was added to distilled water as one of the initial ingredients, together with glycerol. All other steps and concentrations were the same as in the sol-gel method. A schematic image of the different methods is presented in Figure 1.

A 3-2-2 factorial experiment for the sol-gel-ma method with factors maleic anhydride, sugar, and glycerol was performed to optimize preparation conditions and to determine the minimum amount of maleic anhydride needed. All of the concentrations are shown in Table 1, demonstrating the randomized treatments used. This experiment is further referred to as “optimization”, and concentrations of 7% (as used in former studies) are referred to as low, while concentrations of 14% (doubled amount) are referred to as high concentrations. Preliminary experiments showed that only films prepared with citric acid had a good appearance, therefore it was concluded that citric acid is necessary for film formation in order to induce pectin gelation. 

### 2.4. Characterization of Films

#### 2.4.1. Thickness

The thickness of films was measured by a constant-load micrometer. Two measurements were taken of each specimen and the average was reported (mm).

#### 2.4.2. Mechanical Testing

Mechanical testing was performed according to ISO 527 using a Tinius Olsen H10KT tensile tester and data was analyzed with the QMat software package. A moving cross-head was used to pull the specimens with a load cell of 100 N and a velocity of 10 mm/sec. Tensile strength (MPa) and elongation at maximum tensile strength (%) were measured for 5 specimens and the average was reported. 

#### 2.4.3. Water Vapor Permeability

The water vapor transmission permeability (WVP) was determined according to ASTM E96 [18] with modifications and calculated according to Dayarian et al. [19]. A pre-dried (at 70 °C) glass container was filled with 20 ml distilled water, covered with the bio-based films, and sealed with paraffin film. The distance between the film and water was 16 mm. The weight of the whole system was recorded at 0 h and was placed into a desiccator for 5 days. Every 24 h, the weight of the system was recorded to observe the reduction of weight from day 1 to day 5. The mass loss was plotted against time and the slope obtained was used for calculation. The WVP of the films was calculated according to the following equation:

(1) $$WVP=\frac{S*t}{A*\Delta P}$$

where *S* is the slope of the plot (kg/s), *t* is the thickness of the film (m), *A* is the cross-sectional area of the film (m^2^), and Δ*P* is the difference between the vapor pressure of water in the desiccator (assumed to be zero) and inside the container (assumed to be the saturation value at room temperature) (Pa) [19]. The test was performed in duplicates, and averages are reported as the water vapor transmission rate (kg/m Pa s).

#### 2.4.4. Interaction with Water

An interaction-with-water test for selected specimens was performed according to Perotto et al. [6], with slight modifications. A specified size (30 mm × 15 mm) of pre-dried film (for 1 h at 70 °C) was immersed in 20 ml distilled water for 30 min and then dried for 1 h at 70 °C, and the weight loss was calculated by the gravimetric method. The measurements were performed in duplicates and the results are reported as weight loss (%).

#### 2.4.5. Morphology

Surface and transversal morphology was studied by field emission scanning electron microscopy (FE-SEM) (Zeiss, Sigma, Jena, Germany) imaging. For surface visualization, films were attached to a carbon tape and covered with gold. For transversal visualization, cross-sectional images of films were taken as follows: films were immersed in liquid nitrogen for one minute before they were broken and immediately attached to a carbon tape and covered with gold. Photomicrographs were taken at 500, 1.00, 5.00, and 10.00 K × magnifications using an accelerating voltage of 10.00 and 20.00 kV.

#### 2.4.6. Biodegradation Test

Biodegradation of the films was measured by anaerobic digestion. The test was performed according to a previous study [20]. In brief, the films were incubated in 120 mL glass bottles in thermophilic conditions (55 °C) for 30 days. The working volume of the reactors was 50 mL (containing 35.5 mL inoculum with distilled water) and each bottle contained 0.15 g of film volatile solids (VS). The inoculum was obtained from Borås Energi & Miljö AB (Borås, Sweden), a large-scale thermophilic biogas plant. Samples were taken at specified times and analyzed for gas composition with a gas chromatograph (Clarus 500, Perkin-Elmer, Waltham, MA, USA). 

### 2.5. Statistical Analysis

Statistical analyses were performed using MINITAB^®^ software (version 17.1.0, Minitab Inc., State College, PA, USA). Analysis of variance, ANOVA, using the general linear model, was performed to determine the main effects and interactions of different components in the optimization experiment using a 5% significance level. Model assumptions of homogeneity of residuals were checked by inspection of residual plots during model fitting for tensile strength and elongation. 

## 3. Results and Discussion

Lignocellulosic by-products and waste materials are important participants of the bio-economy: their valuable biopolymer content makes them attractive for the bioplastics industry, which at the same time helps address waste handling issues. An example of such a case is bio-based film prepared from orange waste, demonstrated in a previous study [8], which had some holes in its structure. The goal of this study was to modify the film formation method in order to develop a uniform film structure with no holes and in which the adhesion of polymeric components is promoted. 

### 3.1. Effect of Pectin Gelling and Incorporation of Maleic Anhydride on Properties of Films from Orange Waste

The hypothesis that a more coherent film structure would result in the reduction or even elimination of holes in the orange waste film was tested first by the improvement of pectin gelling, followed by the addition of maleic anhydride into the film.

#### 3.1.1. Appearance

Films prepared by the original method had some holes present in the structure, which could be eliminated by a rotary movement during drying; however, that method resulted in uneven thicknesses [8] and required more energy. Inducing pectin gelling prior to casting improved pectin binding and resulted in a reduced number of holes of a smaller size. By applying this method, the use of rotary movement was not necessary and films had even thicknesses. Because some smaller holes were still present, however, further improvements were necessary and maleic anhydride was used.

When maleic anhydride was used for film preparation, the holes completely disappeared and films with uniform structure were obtained.

#### 3.1.2. Morphology

**Figure 2:**
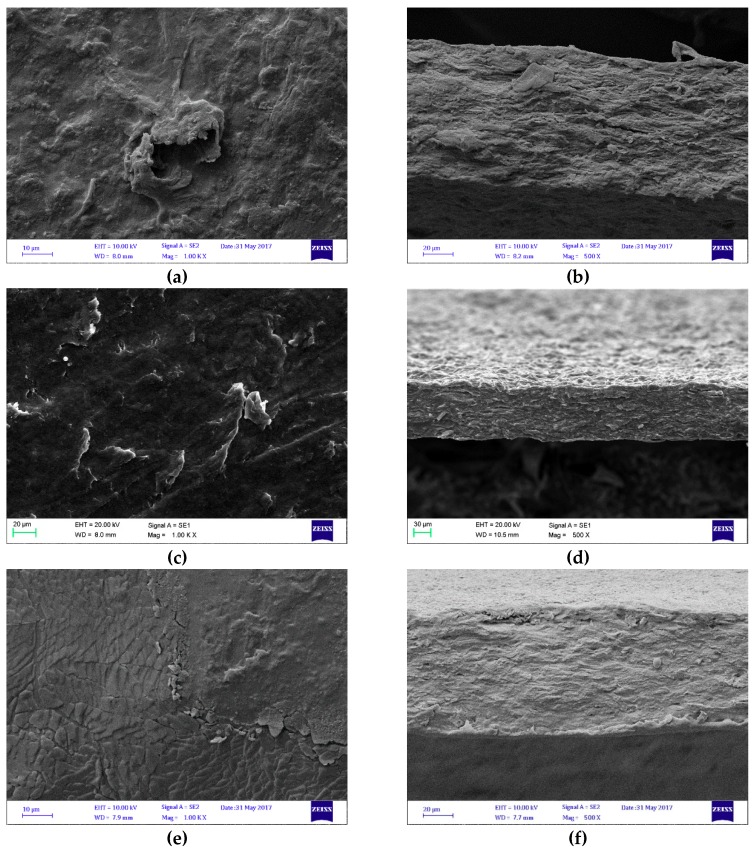
Field emission scanning electron microscopy (FE-SEM) micrographs of the film surface and cross-sectional images prepared with the sol-gel method (**a**,**b**); sol-gel-ma (1.56% maleic anhydride) (**c**,**d**); and sol-gel-ma (25% maleic anhydride) (**e**,**f**). The magnification of surface images was 1.00 K × and that of cross-sectional images was 500×.

On a microstructural level, according to FE-SEM images, a more compact film construction was achieved using maleic anhydride compared to the sol-gel method (Figure 2). The micrographs also seem to show smoother surface morphology with increasing maleic anhydride concentration (Figure 2a,c,e). 

The smoother and more compact structure is probably the direct effect of the additional ester bonds between grafted maleic anhydride intermediates and cellulose [21] and/or pectin [22] and/or glycerol [23]. Since the bio-based films prepared in this study represent a complex mixture of biopolymers (e.g. pectin, cellulose, hemicellulose) and other hygroscopic substances such as glycerol and sugar, which contain OH groups and can function as plasticizers [24], it is difficult to determine which component reacted most with maleic anhydride. 

As the introduction of maleic anhydride improved the appearance and structure of the films in general, the next step was to find the optimal concentration of maleic anhydride and other components in the films. Aiming to reduce the amount of chemicals used, the goal was to find the minimum concentration of maleic anhydride in which a film with improved characteristics is obtained.

#### 3.1.3. Thickness and Mechanical Properties

The thicknesses of the films prepared by the original and sol-gel methods ranged between 0.098 and 0.135 mm, with an average value of 0.115 ± 0.010 mm (standard deviation). The mechanical properties of the films made by the original and sol-gel methods were not significantly different, resulting in a tensile strength of 21.08 ± 7.78 and 21.69 ± 8.72 MPa and elongation of 3.67 ± 1.80 and 4.83% ± 1.79%, respectively. In general, the addition of maleic anhydride into the films resulted in lower tensile strength and higher elongation compared to those of the sol-gel method (Table 2). At higher concentrations of maleic anhydride (≥ 1.56%), the elongation of the films increased as maleic anhydride content was reduced. This fact contradicts the hypothesis that maleic anhydride would act as a cross-linker in the pectin-cellulose system because cross-linking generally increases the strength and stiffness and reduces elongation. Therefore, instead of a cross-linking agent, maleic anhydride could rather be considered as a compatibilizer that improved film formation and also provided a significant plasticizing effect. The plasticizing effect of maleic anhydride on cellulose diacetate has also been confirmed before [23].

### 3.2. Optimization of Maleic Anhydride, Sugar, and Glycerol Concentrations to Enhance the Film Properties

In order to reduce the amount of maleic anhydride needed for the production of the films, and in continuation of the preliminary experiment (Section 3.1), the effect of maleic anhydride was also studied at levels of 0.78% and 0.39%. At the same time, the effect of sugar and glycerol at elevated levels (14%) and the interaction between the components affecting the mechanical properties was also studied.

#### 3.2.1. Morphology

**Figure 3:**
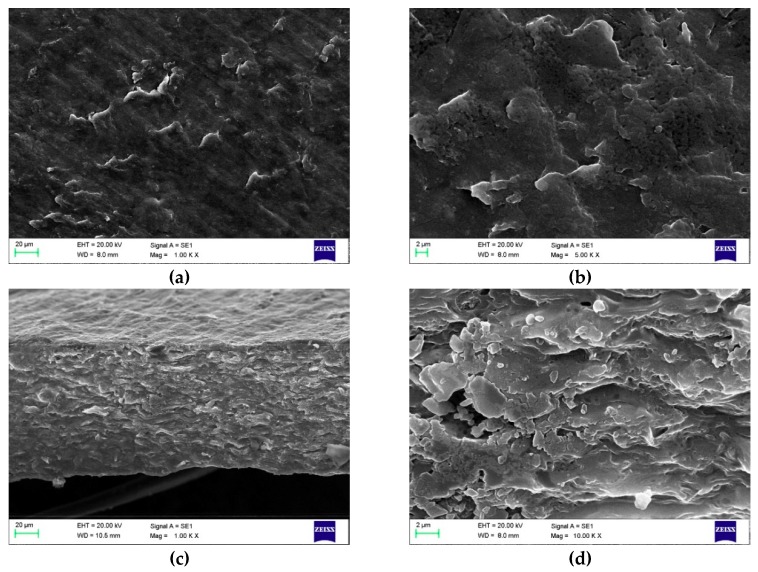
E-SEM micrographs of film surfaces with a magnification of 1.00 K × (**a**) and 5.00 K × (**b**) and cross-sectional images with a magnification of 1.00 K × (**c**) and 10.00 K × (**d**) containing the highest concentration of glycerol (14%) prepared according to the sol-gel-ma method (maleic anhydride and sugar concentrations were 0.39% and 7%, respectively).

**Figure 4:**
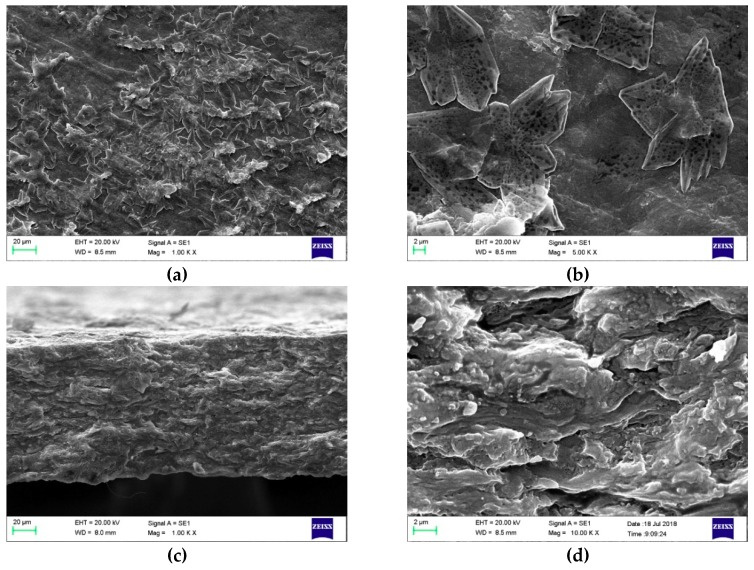
FE-SEM micrographs of film surfaces with a magnification of 1.00 K × (**a**) and 5.00 K × (**b**) and cross-sectional images with a magnification of 1.00 K × (**c**) and 10.00 K × (**d**) containing the highest concentration of sugar (14%) prepared according to the sol-gel-ma method (maleic anhydride and glycerol concentrations were 0.39% and 7%, respectively).

**Figure 5:**
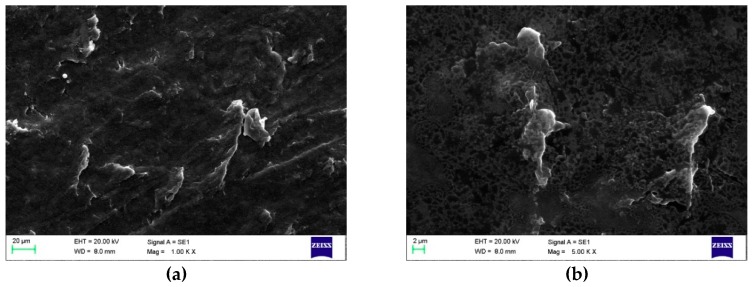

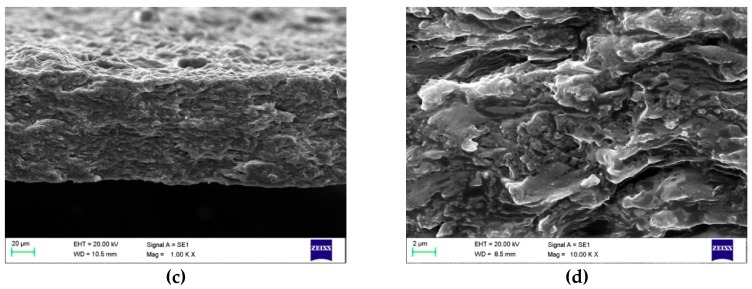
FE-SEM micrographs of film surfaces with a magnification of 1.00 K × (**a**) and 5.00 K × (**b**) and cross-sectional images with a magnification of 1.00 K × (**c**) and 10.00 K × (**d**) containing the highest concentration of maleic anhydride (1.56%) prepared according to the sol-gel-ma method (both glycerol and sugar concentrations were 7%).

The morphology of films containing the most glycerol, sugar, or maleic anhydride with the lowest concentrations of the other two components were studied and are shown in Figure 3, Figure 4 and Figure 5. 

The surface images of the films containing the highest amount of glycerol (Figure 3a,b) show softer, more curvy edges of particles, and a fluffier cross-section image can also been seen (Figure 3c,d). The fluffiness of the structure can be explained by the plasticizing effect of the glycerol, in which the small molecular weight compound is pushing the molecules slightly farther apart to enhance the mobility and the softness of the structure.

The surface images of films containing the highest concentrations of sugar show elegantly arranged flower-like patterns (Figure 4a,b). These patterns may be from the formation of sugar crystals when sugar is present at a high concentration in the solution. A possible explanation could be that as water evaporates during the drying of films, the solution becomes more saturated with sugar and the molecules continuously come out to the surface to re-arrange themselves into crystals [25]. This phenomenon was only observed when sugar was added to the mixture at a high concentration, which may be due to excess sugar in the system not taking part in the film-forming reactions. However, this needs to be confirmed in future studies. There is no evidence that this phenomenon is the re-crystallization [26] of sugar and of how it could have a positive impact on elongation. However, different sugars are known to act as plasticizers in biopolymer films [24]. 

Films that contain the highest concentration of maleic anhydride have a leather-like and denser appearance on the surface (Figure 5a,b), and they represent a more compact structure when the cross-sectional images are observed (Figure 5c,d). The denseness of the films could be the result of the additional bonds formed between maleic anhydride and other molecules.

Attention was also paid to films that contain high concentrations of both, sugar and glycerol at various maleic anhydride levels. Specifically, the possible morphological differences between specimens containing various maleic anhydride levels have been investigated. The same morphology that was observed for high sugar and high glycerol content can also be observed on all of these images: floral patterns are formed on a fluffy background, representing the excess sugar and the loosening effect of glycerol. The effect of maleic anhydride, however, is difficult to observe when glycerol and sugar are used in high concentrations. Because of the high number of FE-SEM images, these images are available as supplementary material (Figure S1).

#### 3.2.2. Thickness of Films

The thickness of the films prepared in the optimization experiment ranged between 0.095 and 0.139 mm, with an average value of 0.120 ± 0.010 mm (standard deviation), which was similar to preliminary experiments (Section 3.1.3). The use of different concentrations of glycerol had a significant positive effect on film thickness (p = 0.000), while sugar had small but non-significant effect (p = 0.067), and maleic anhydride had no effect (p = 0.620). A clear trend could not be observed, however.

#### 3.2.3. Mechanical Properties

**Figure 6:**
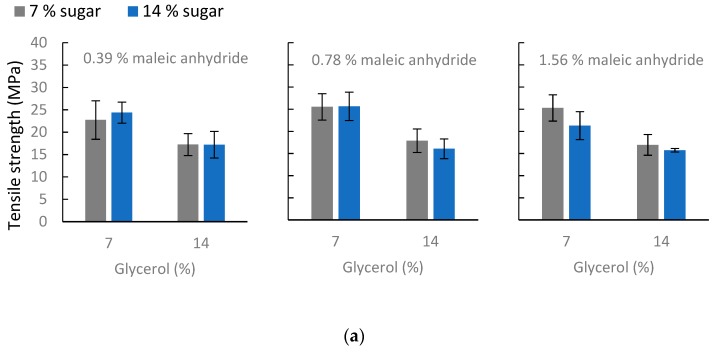

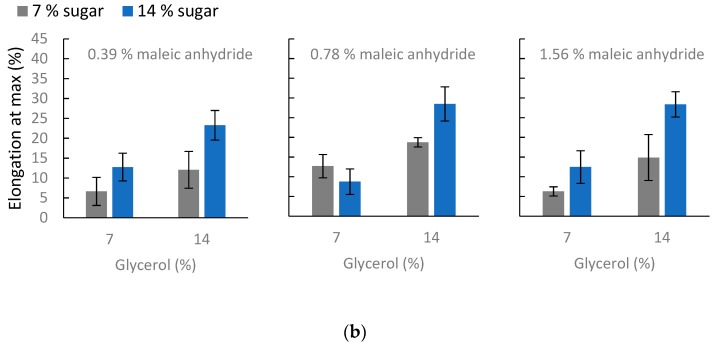
Mean values of tensile strength (**a**) and elongation at max (**b**) of films from the optimization experiment for different maleic anhydride levels. The X-axis represents the concentrations (%) of glycerol (7 and 14); and the grey and blue colors represent 7 and 14 % sugar levels, respectively, used in the films. Individual standard deviations were used to calculate the intervals.

Glycerol appears to have a significant negative effect (coefficient −3.64, p = 0.000) on the tensile strength (Figure 6a) when increased from low (7%) to high (14%) concentration, as was expected. Sugar and maleic anhydride (p = 0.213 and p = 0.231, respectively) did not have any significant effect on tensile strength. 

The elongation values are clearly more complicated, as a number of mechanisms may be involved in the plasticization of bioplastics with low molecular weight compounds [27]. Elongation was significantly affected by glycerol (the reverse effect of that for tensile strength, as expected) and sugar (coefficient 5.52, p = 0.000 and coefficient 3.57, p = 0.000, respectively). Their interaction also had a significant positive effect on elongation (coefficient 2.18, p = 0.000). This result, in the observed pattern of high glycerol and high sugar levels, gives the highest elongation for all maleic anhydride levels (Figure 6b). The effect of maleic anhydride concentration on elongation is made up of a main effect and varying interaction effects. The maleic anhydride main effect on elongation and the maleic anhydride*sugar interaction are both significant. When maleic anhydride was used at a low (0.39%) concentration, the effect on elongation was negative (coefficient −1.76, p = 0.013). When maleic anhydride was used at a concentration of 0.78%, the effect was positive (coefficient 1.72, p = 0.015), and when it was used at a concentration of 1.56%, the effect was also positive but smaller than at the middle level (0.78%) (coefficient 0.046, p = 0.946). The maleic anhydride*sugar interaction was most noticeable when 0.78% maleic anhydride was used (coefficient 2.18, p = 0.03). From the general linear model, significant positive effects were found for high (14%) sugar and glycerol levels, and a positive sugar*glycerol interaction for this combination was also found. Furthermore, the modelled values and 95% confidence intervals calculated for the different maleic anhydride concentrations at the highest levels of glycerol and sugar (14%) were 24.16 (21.2, 27.23), 27.24 (24.18, 30.30), and 28.71 (25.65, 31.77) for 0.39%, 0.78% and 1.56% maleic anhydride, respectively. 

#### 3.2.4. Water Vapor Permeability and Interaction with Water

In general, all films were flexible and uniform, and among them, four were selected randomly in regards to the experimental design (containing 1.56%, 0.78%, 0.38%, and 1.56% maleic anhydride, 14%, 7%, 14%, and 14% sugar, and 14%, 14, 7%, and 7% glycerol, respectively) for water vapor permeability and water interaction testing. The selected specimens also had the most uniform structure, based on observation. 

Low water vapor permeability is often a requirement, e.g., for food packaging materials. The lower the value, the higher is the resistance to water vapor. The water vapor permeability of bio-based films prepared in the optimization experiment, regardless of preparation method, had a mean value of 1.19 (± 0.08) × 10^−13^ kg/m Pa s, which can be compared with the WVP of fungal biomass-reinforced pectin films of 2.35 × 10^−13^ kg/m Pa s [28] and edible films from alginate-acerola reinforced with cellulose whiskers of 1.67 (± 0.42) × 10^−13^ kg/m Pa s [29]. Both of these studies reported lower WVP values when the reinforcement load increased. The lowest value was obtained when fungal biomass load was 35 wt % [28] and when cotton cellulose whiskers were used at 15 wt % [29]. 

These results are, however, higher than those of polysaccharides-based locust bean gum films prepared by solution casting with different plasticizers (polyethylene glycol 200, glycerol, propylene glycol, and sorbitol), which range between 1.2 and 2.6 × 10^−14^ kg/m Pa s [30], meaning a lower resistance to the passage of water vapor.

All of the specimens used for the test lost more than half of their dry weight, 56.15% ± 3.37%. In a study performed by Perotto et al. [6], bioplastic films fabricated from vegetable wastes showed approximately 35% solubility after 108 h of immersion in water, however. These results indicate the unfortunate feature of polysaccharides-based bioplastics: their hydrophilic nature.

### 3.3. Biodegradation

A concentration of 1.56% maleic anhydride was the highest used in the optimization experiment; therefore, it was used for the study of biodegradation. The maximal biodegradation rate of orange waste films made with the sol-gel and sol-gel-ma methods was 63% and 52%, respectively (Table 3). According to a previous study [8], orange waste films produced with the original method showed 90% biodegradability within approximately 15 days. The reason for a lower degradation rate in this study could be that (1) the effect of maleic anhydride present in the structure may make the film more difficult for the microorganisms to degrade (however, sol-gel films did not contain maleic anhydride); (2) the inoculum was not the same as in the previous study [8]. The available microbial community in a digester, used as inoculum, depends on the actual composition of the food waste available, which cannot be guaranteed to be the same. Therefore, in this case, although the same experiment was performed with the same parameters and with the same source of inoculum, it resulted in lower biodegradation rates.

## 4. Conclusions

The present study recognized the complexity of the different components used in the pectin–cellulose system and that the interpretation of the contrasting behavior can be rather difficult. The enhancement of pectin gelling prior to film casting had a positive effect on the film structure, reducing the number of holes present. The positive effect of maleic anhydride was further shown to eliminate holes in orange waste films via an improved structure with a smooth and uniform surface. For this effect, maleic anhydride was only necessary at very low concentration (0.39%). On the other hand, statistical analyses proved that the tensile strength of the films depended on glycerol content, and elongation was mainly dependent on both glycerol and sugar. The interaction between these was also significant. The highest elongation was reached at the highest levels of sugar and glycerol for both 0.78% and 1.56% maleic anhydride concentrations; therefore, to reach the highest elongation values, the use of maleic anhydride at a concentration of 0.78% was satisfactory. This observation led to the rejection of the hypothesis that maleic anhydride would act as a cross-linking agent; therefore, it was concluded that maleic anhydride in the orange waste films acts as a compatibilizer.

Because orange waste film is biodegradable in anaerobic conditions, it could be suitable as a future material for collecting bag of the organic fraction of municipal solid waste; however, the hydrophilicity of the material needs to be improved. The degradation test performed here contained 1.56% maleic anhydride, but results of other analyses showed that as little as 0.39% is sufficient for a uniform material. The degradation of orange waste film containing 0.39% maleic anhydride would probably be acceptable in a biogas plant, but to state this, exact measurements would be needed. 

## Figures and Tables

**Table 1:** Different concentrations (*w*/*w* %) of maleic anhydride, sugar, and glycerol in the optimization experiment. Citric acid concentration was constant at 1% (*w*/*v*).

Maleic Anhydride	Sugar	Glycerol
1.56	7	7
0.39	7	14
0.78	14	7
1.56	7	14
1.56	14	14
0.78	7	7
0.78	14	14
0.39	7	7
0.39	14	14
0.78	7	14
0.39	14	7
1.56	14	7

**Table 2:** Mean values of tensile strength (TS) and elongation at max (E) of films prepared in the preliminary experiment using different concentrations of maleic anhydride (MA) and sugar (S).

method	MA (%)	S (%)	TS (MPa)	st. dev.	E (%)	st. dev.
original ^1^	0	0	31.67	4.21	3.02	0.62
original ^2^	0	0	34.76	2.64	4.23	1.71
original	0	0	21.08	7.78	3.67	1.80
sol-gel	0	7	21.69	8.72	4.83	1.79
sol-gel-ma	100	7	13.28	1.71	11.03	0.55
sol-gel-ma	50	7	12.51	1.97	14.43	3.00
sol-gel-ma	25	7	10.58	3.18	18.60	3.55
sol-gel-ma	12.5	7	5.79	0.76	14.53	3.83
sol-gel-ma	6.25	7	9.80	1.63	20.70	1.85
sol-gel-ma	3.13	7	10.00	2.14	20.23	2.30
sol-gel-ma	1.56	7	9.75	2.13	21.98	3.25

^1^ Films dried in an oven according to Bátori *et al*. [8]. ^2^ Films dried in a rotary incubator according to Bátori *et al*. [8].

**Table 3:** Biodegradation rate (%) of bio-based films produced by the sol-gel and sol-gel-ma methods, containing 1.56 *w*/*w* % maleic anhydride.

Days	0	3	6	9	12	15	20	25	30
**Sol-gel**	0	22	40	59	60	63	59	58	59
**Sol-gel-ma**	0	22	39	53	51	52	49	49	49

## Supplementary Materials

The following are available online at https://www.mdpi.com/2073-4360/11/3/392/s1, Figure S1: FE-SEM micrographs of film surfaces with a magnification of 1.00 K × (a) and 5.00 K × (b) and cross-sectional images with a magnification of 1.00 K × (c) and 10.00 K × (d) containing the highest concentrations of glycerol and sugar and the lowest concentration of MA (0.39%); film surfaces with a magnification of 1.00 K × (e) and 5.00 K × (f) and cross-sectional images with a magnification of 1.00 K × (g) and 10.00 K × (h) containing the highest concentrations of glycerol and sugar and the middle level of MA concentration (0.78%); film surfaces with a magnification of 1.00 K × (i) and 5.00 K × (i) and cross-sectional images with a magnification of 1.00 K × (k) and 10.00 K × (l) containing the highest concentrations of glycerol and sugar and the highest concentration of MA (1.56%).
