# Supplementary material for: The Effect of Glycerol, Sugar, and Maleic Anhydride on Pectin-Cellulose Thin Films Prepared from Orange Waste
Source: Polymers (Basel). 2019 Feb 27;11(3):392. doi: 10.3390/polym11030392 (PMC6474150; doi:10.3390/polym11030392)
Supplement: Supplementary file 1 [file polymers-11-00392-s001.pdf]

Supplementary material 1

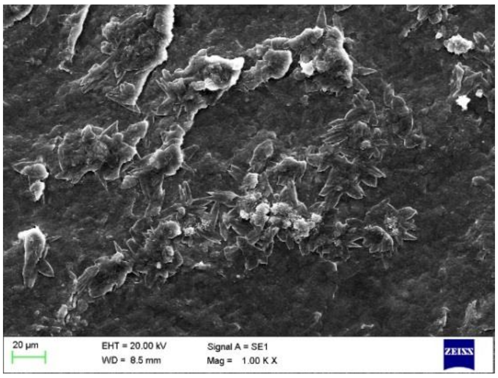

(a)

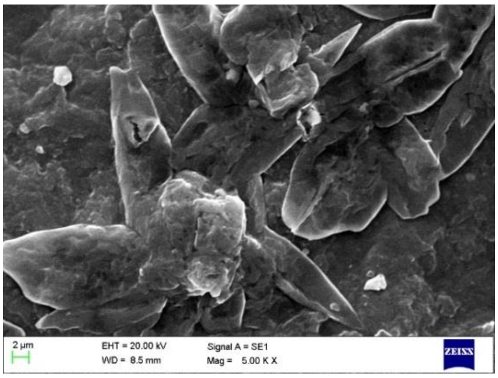

(b)

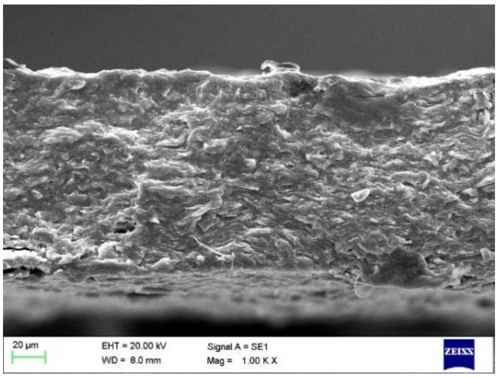

(c)

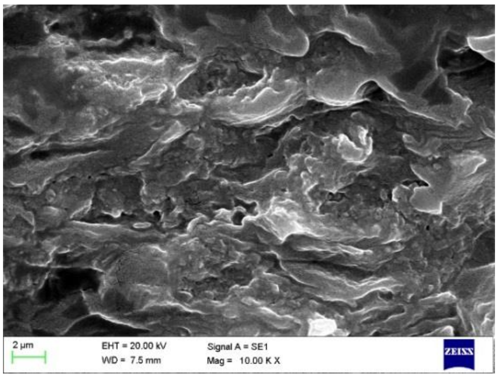

(d)

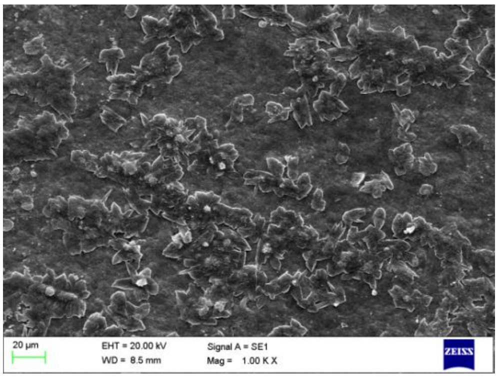

(e)

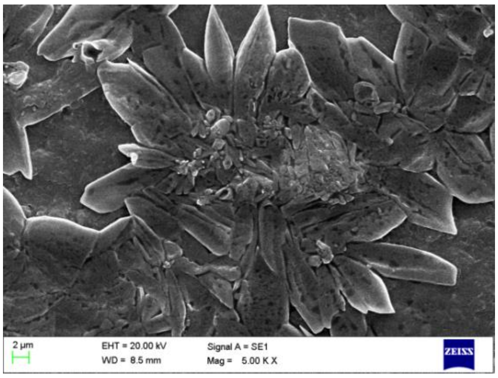

(f)

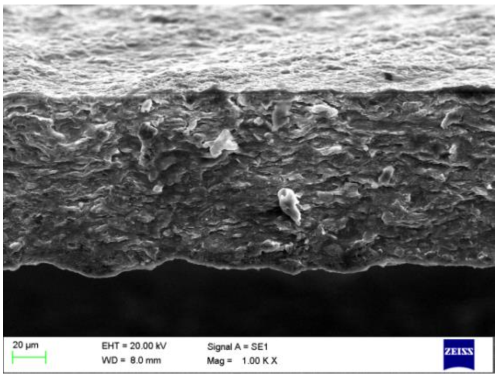

(g)

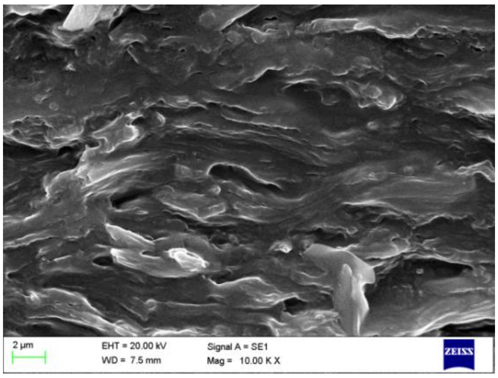

(h)

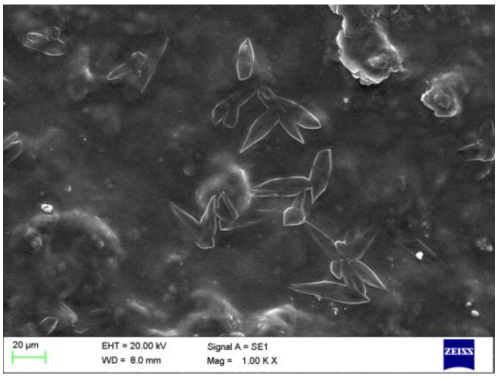

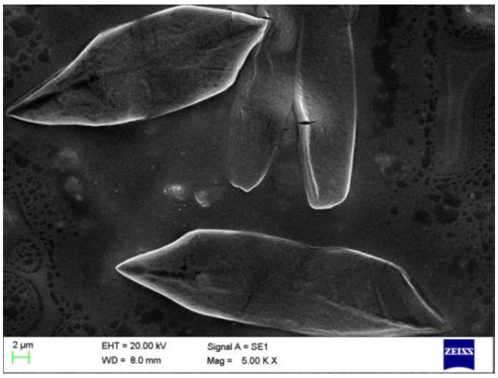

(i) **(j)**

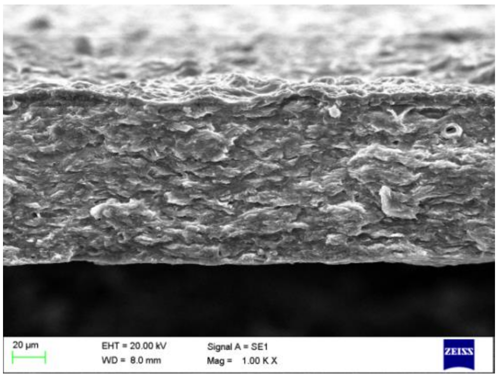

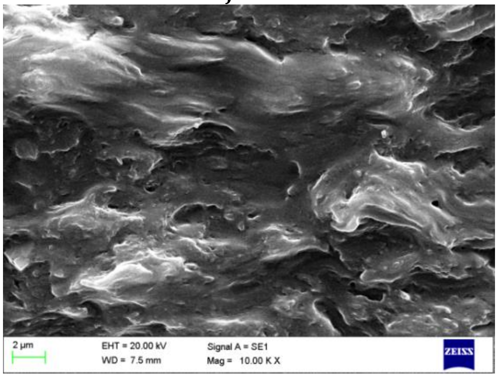

(k) **(l)**

Figure S1. FE-SEM micrographs of film surfaces with a magnification of 1.00 K × (**a**) and 5.00 K × (**b**) and cross-sectional images with a magnification of 1.00 K × (**c**) and 10.00 K × (**d**) containing the highest concentrations of glycerol and sugar and the lowest concentration of MA (0.39%); film surfaces with a magnification of 1.00 K × (**e**) and 5.00 K × (**f**) and cross-sectional images with a magnification of 1.00 K × (**g**) and 10.00 K × (**h**) containing the highest concentrations of glycerol and sugar and the middle level of MA concentration (0.78%); film surfaces with a magnification of 1.00 K × (**i**) and 5.00 K × (**i**) and cross-sectional images with a magnification of 1.00 K × (**k**) and 10.00 K × (**l**) containing the highest concentrations of glycerol and sugar and the highest concentration of MA (1.56%).
